# Effect of Acute Hypercapnia on Outcomes and Predictive Risk Factors for Complications among Patients Receiving Bronchoscopic Interventions under General Anesthesia

**DOI:** 10.1371/journal.pone.0130771

**Published:** 2015-07-06

**Authors:** Qinghao Cheng, Jieli Zhang, Hongwu Wang, Rujin Zhang, Yun Yue, Lei Li

**Affiliations:** 1 Department of Anesthesiology, China Meitan General Hospital, Beijing 100028, China; 2 Minimal Invasive Tumor Therapy Center, China Meitan General Hospital, Beijing100028, China; 3 Department of Anesthesiology, Beijing United Family Hospital, Beijing 100015, China; 4 Department of Anesthesiology, Beijing Chaoyang Hospital, Capital Medical University, Beijing 100020, China; University of Pennsylvania, UNITED STATES

## Abstract

**Background:**

The aim of this study is to investigate the effect of acute hypercapnia on surgery outcomes among patients receiving bronchoscopic interventions under general anesthesia. Furthermore, independent predictive factors for surgery complications were analyzed.

**Method:**

A total of 323 patients with airway stenosis were enrolled in this retrospective study. Each patient underwent interventional rigid bronchoscopy under general anesthesia. Arterial blood gas (ABG) was measured intraoperatively. In light of PaCO_2_ levels in ABG, patients were divided into three groups: Group C (control) (PaCO_2_:≤ 60 mmHg), Group M (moderate) (PaCO_2_:61–100 mmHg), and Group S (severe) (PaCO_2_: >100 mmHg). Parameters, including PaO_2_ levels and recovery delays, were compared across three groups. Complications among patients receiving bronchoscopic interventions were evaluated as well. Independent predictive factors for surgery related complications were analyzed by multivariable regression method.

**Results:**

Significant differences in weight (*p*=0.04), ASA IV (*p*=0.008), dyspnea index (*p*=0.003),COPD (*p*=0.02), dynamic airway collapse (*p*=0.002), severe stenosis severity (*p*=0.02), and stenosis locations among three groups were observed. Mild (PaCO_2_:~60 mmHg) to moderate (PaCO_2_:60–100 mmHg) hypercapnia was not associated with delayed recovery, whereas severe hypercapnia (PaCO_2_:>100 mmHg) was associated with delayed recovery, as well as declined PaO_2_ (*p*=0.00) and elevated blood glucose levels (*p*=0.00). The complications of bronchoscopic interventions included postoperative congestive heart failure (14 cases, 4.3%), tracheorrhagia (8 cases, 2.5%), delayed recovery (19 cases, 5.9%), and transfers to ICU after surgery (10 cases, 3.1%). The multivariable regression analysis showed that procedure duration (*p*=0.003), lobectomy (*p*=0.007), dynamic airway collapse (*p*=0.01), severe bronchial stenosis (*p*=0.01) and hypercapnia (*p*=0.02) were independent predictive factors for surgery related complications.

**Conclusions:**

Acute hypercapnia lower than 100 mmHg was not associated with detrimental consequences, whereas severe hypercapnia (PaCO_2_: >100 mmHg) was associated with lower levels of PaO_2_. Hypercapnia was an independent predictive factor for bronchoscopic intervention complication, which may help physicians to optimize the therapeutic choices.

## Introduction

Various pathologies could lead to air obstruction. Over one-fifth of lung cancer cases progress to airway obstruction [1,2]. Bronchoscopic procedures, which aim to reopen the airway and remove intraluminar malignancy, are typically performed under local anesthesia [3]. Numerous technologies and tools, such as stent, cryocanalization, electrocautery, and microdebrider, are developed for bronchoscopic interventions [1]. These bronchoscopic interventions remarkably increase the success rate of removing tumors and significantly improve the quality of patient life [4]. Accordingly, these procedures require intensive anesthesia, for which general anesthesia is suitable.

Permissive hypercapnia, as a practical ventilation strategy, is widely used to improve patient outcomes. Through this approach, tidal volume and alveolar ventilation are reduced, resulting in less ventilator-associated lung injuries. Recent animal study shows that mild to moderate hypercapnia (PaCO_2_:60–100 mmHg) has a neuroprotective effect on rats with a transient global cerebral ischemia–reperfusion injury [5]. In clinical scenarios, acute therapeutic hypercapnia is allowed in thoracic surgery, during which no serious consequences are observed [6,7]. However, the effect of permissive or acute hypercapnia on bronchoscopic intervention is not yet determined.

In this study, we retrospectively evaluate the effect of acute hypercapnia on patients receiving bronchoscopic interventions under general anesthesia. Furthermore, incidences of complication are reviewed.

## Materials and Methods

### Study design and objectives

A total of 323 patients who underwent bronchoscopic interventions from March 2010 to March 2013 were enrolled in this study. A bronchoscopic intervention team, consisting of surgeons and anesthesiologists, selected the cases for surgery. Video-assisted rigid bronchoscopy, combined with electric flexible fiber bronchoscopy, was performed in each patient, and the complications were reviewed through medical records.

The study was approved by the ethical committee of the China Meitan General Hospital. Written consent was obtained from each participant.

### Anesthetic settings, induction, and maintenance

Patients were categorized into three groups based on the PaCO_2_ levels in arterial blood gas (ABG): Group C (control) (PaCO_2_:≤ 60 mmHg), Group M (moderate) (PaCO_2_:61–100 mmHg), and Group S (severe hypercapnia) (PaCO_2_: >100 mmHg).

Patients were routinely monitored by electrocardiogram, non-invasive blood pressure, and pulse oximetry. Anesthesia was induced along with fast-recovery drugs, i.e.,propofol, remifentanil, rocuronium, or succinylcholine chloride. Propofol and remifentanil were intravenously infused to obtain deep anesthesia levels with no oropharyngeal and cough reflexes. During the bronchoscopic procedures, patients were anesthetized to a state with no body movement. Patients who could not be weaned off mechanical ventilation after completing the surgery were transferred to the ICU for further recovery.

High-frequency jet ventilation (HFJV) was connected to the lateral port of a rigid bronchoscope (Jiangxi Teli Medical Instruments, China). Initial settings were as follows: respiratory rate: 16–24 bpm; driving pressure:42.67 Psi; and inspiratory fraction: 50%. Inspired oxygen fraction (FiO_2_) was set at 100% during the entire intervention. When PaCO_2_ was higher than 100 mmHg or when SpO_2_ was lower than 90%, HFJV was discontinued and manual ventilation was commenced. Once PaCO_2_ decreased below 80mmHg, HFJV was continued as long as the patient condition allowed it.

### Measurements and monitoring

ABG analysis was performed (blood gas analyzer, GEM Premier 3000, Instrumentation Laboratory, Lexington, MA, USA) on the blood samples drawn during the bronchoscopic procedures.

### Bronchoscopic interventional procedures

Rigid bronchoscope (Storz, Germany) was intubated after the patients were anesthetized. Electric flexible bronchoscope (Pantex and Olympus, Japan) was inserted through a rigid tube. Multiple procedures, including electric loops, cryoprobes, argon plasma coagulation (APC), endobronchial ultrasound, stents, and radioactive iodine 125-particle implantation, were performed to recanalize obstructed lumens, kill tumor cells, and relieve apnea symptom [1].

### Data collection and statistics analyses

Demographics, complications, and surgical results were retrospectively collected from the medical records, which included anesthesia types, surgical procedures, and medications.

Statistical analyses were performed using SPSS version 11.0 (SPSS Inc., Chicago, USA). Data were expressed as mean ± standard deviation. The differences of measurable data among the three groups were compared through one-way ANOVA. Furthermore, the countable data among the three groups were compared by chi-square test. Multivariate regression analysis was used to detect independent predictive factors for poor operation-related outcomes. Logistic regression was used to calculate odd ratios. A level of *P*<0.05 was considered statistically significant.

## Results

### Patient features in three groups

A total of 323 patients were included in this study. The characteristics of each group are displayed in Table 1. No significant differences in age, gender, height, ASA classification (I–III) scores, smoking habits, Karnofsky performance status (KPS), and comorbidities (diabetes mellitus, coronary heart disease, hypertension, bronchiectasis, lobectomy, andatelectasis) were observed across the three groups. No significant differences in weight (*p* = 0.04), ASA IV (*p* = 0.008), dyspnea index (*p* = 0.003), COPD (*p* = 0.02), dynamic airway collapse (*p* = 0.002), and stenosis severity (*p* = 0.02) were seen among the three groups (Table 1). The locations of stenosis in the trachea or in the bronchus differed across the three groups (Table 1).

**Table 1:** Description of patient characteristics in three groups.

	Group C	Group M	Group S	*P*
	(*N* = 83, 25.7%)	(*N* = 193, 59.8%)	(*N* = 47, 14.6%)	value
Age,(years)	58.4±11.3	55.9±16.0	57.5±14.0	0.40
Male,(*n*)	63(75.9)	136(70.5)	29(61.7)	0.23
Weight,(kg)	63.8±11.3	63.7±12.3	68.8±16.7	0.04
Height,(cm)	168.3±7.3	166.3±13.7	166.3±7.2	0.40
Smoker,(*n*)	48(57.8)	111(57.5)	27(57.4)	0.90
**ASA physical status scale**				
ASA I	27(32.5)	55(28.5)	10(21.3)	0.39
ASA II	24(28.9)	48(24.9)	11(23.4)	0.72
ASA III	19(22.9)	40(20.1)	7(14.9)	0.55
ASA IV	13(15.7)	50(28.5)	19(40.4)	0.008
Dyspnea index	2.1±1.1	2.4±1.2	2.8±1.2	0.003
**Comorbidities**				
Diabetes mellitus	9(10.8)	20(10.4)	7(14.9)	0.67
Chronic heart disease	9(10.8)	14(7.3)	7(14.9)	0.23
Hypertension	25(30.1)	40(20.7)	11(23.4)	0.24
Bronchiectasis	2(2.4)	3(1.6)	2(4.3)	0.51
COPD	9(10.8)	26 (13.5)	13(27.7)	0.02
Lobectomy	4(4.8)	15(7.8)	8(17.0)	0.05
Atelectasis	6(7.2)	16(8.3)	4(8.5)	0.95
**Pathology**				
Tumor	75(90.4)	155(80.3)	31(66.0)	0.003
Non-tumor	8(9.6)	38(19.7)	16(44.0)	0.003
**Anatomy**				
Intrinsic Stenosis	70(84.3)	170(88.1)	36(76.6)	0.13
ExtrinsicCompression	12(14.5)	18(9.3)	5(10.6)	0.45
DAC	1(1.2)	5(2.6)	6(12.8)	0.002
**Locations**				
Trachea	11(13.3)	57(25.9)	15(31.9)	0.01
Bronchus	62(74.7)	60(31.1)	10(21.3)	0.00
Trachea+Bronchus	10(12.0)	76(39.4)	22(46.8)	0.00
**Severe stenosis**	11(13.3)	31(16.1)	15(31.9)	0.02

Data are presented as mean± standard deviation (median, range) or as a number (percentage).

Severe stenosis: diameter of trachea < 6 mm; the obstruction is above 90% of the main bronchus in a cross-sectional area. KPS: Karnofsky performance status. DAC: dynamic airway collapse.

### Procedure duration and weaning time

The procedure duration (*p* = 0.00), post-surgery weaning time (*p* = 0.00), and operation room stay (*p* = 0.00) significantly differed among the three groups (Table 2). Group S indicated the longest durations of endoscopic procedures, post-surgery weaning time, and operation room stay for recovery. Mild to moderate (PaCO_2_: 60–100 mmHg) hypercapnia was not associated with delayed recovery, whereas severe hypercapnia (PaCO_2_: >100 mmHg) was associated with delayed recovery, as well as lengthy post-surgery weaning time and operation room stay. The PaO_2_ levels gradually decreased in Groups C to S(*p* = 0.00). Group S recorded the highest blood glucose (*p* = 0.00) among the groups.

**Table 2:** Arterial blood gas analyses and surgical complications across three groups.

	Group C	Group M	Group S	*P*
	(*N* = 83, 25.7%)	(*N* = 193, 59.8%)	(*N* = 47, 14.6%)	value
PaCO_2_(mmHg)	49.7±7.4	74.8±10.5	112.5±4.3	0.00
PaO_2_ (mmHg)	293.8±145.8	266.8±139.5	162.0±111.3	0.00
PH	7.35±0.06	7.22±0.06	7.03±0.09	0.00
Glucose (mmol/L)	6.60±1.87	6.98±1.61	9.10±2.74	0.00
Lactase (mmol/L)	0.85±0.39	0.72±0.38	0.90±1.46	0.14
Procedures duration (min)	71.3±32.1	75.4±35.2	104.5±75.4	0.00
Time of ventilation weaning (min)	5.4±2.2	5.8±2.6	8.1±5.1	0.00
Stay in OR after operation (min)	7.3±3.6	9.1±8.2	13.1±11.6	0.00
Hypoxemia	0	3(1.6)	3(6.4)	0.03
Tracheorrhagia	1(1.2)	4(2.1)	3(6.4)	0.16
Delayed recovery	0	12(6.2)	7(14.9)	0.00
Postoperative CHF	1(1.2)	6(3.1)	7(14.9)	0.00
Transfer to ICU	1(1.2)	3(1.6)	6(12.8)	0.00

Data are presented as mean± standard deviation or patient number.

Tracheorrhagia: blood volume above 200mL; Delayed recovery: recovery time >30min; Hypoxemia: arterial oxygen partial pressure (PaO_2_) below 60mmHg; Hypercapnia: arterial CO_2_ partial pressure (PaCO_2_) above 45 mmHg. CHF: congestive heart failure; OR: operation room.

### Surgery related complications

Primary complications included postoperative congestive heart failure (14 cases, 4.3%), tracheorrhagia (8 cases, 2.5%), delayed recovery (19 cases, 5.9%), and transfers to intensive care unit after surgery (10 cases, 3.1%) (Table 2). No procedure-related deaths occurred.

### Predictive factors for complications

In an attempt to identify predictive factors for interventional complications (postoperative congestive heart failure, tracheorrhagia, delayed recovery and transferring to intensive care unit), multivariable regression analysis was conducted. The results showed that procedure duration (*p* = 0.003), lobectomy (*p* = 0.007), dynamic airway collapse (*p* = 0.01), severe bronchial stenosis (*p* = 0.01) and hypercapnia (*p* = 0.02) were independent predictive factors for intervention complications (Table 3).

**Table 3:** Independent predictive factors for surgery complications.

Complications	Independent predictive factor	Odds ratio(95%CI)	*P*
Postoperative congestive heart failure, tracheorrhagia, delayed recovery and transfer to ICU	Procedures duration*	0.079(0.028~0.13)	0.003
	Lobectomy	12.6(4.8~20.3)	0.007
	Dynamic airway collapse	15.6(3.5~27.8)	0.01
	Severe bronchial stenosis	7.56(1.55~13.57)	0.01
	Hypercapnia	3.21(2.3~ 7.6)	0.02

The odds were calculated by logistic regression models. The independent predictive factors, in this analysis includes age, gender, weight, height, smoke status, ASA physical status scale, comorbidities (diabetes, chronic heart disease, hypertension, etc.), pathology, tumor location. Only significant independent predictive factors were listed in the table.

*Indicates that a long procedure time is a risk factor for complications.

## Discussion

Rigid bronchoscopy was first performed in 1947 under local anesthesia. From then, the procedure served as an effective tool in managing central airway obstructions because of variable pathologies. Rigid bronchoscopy can be performed under the conditions of deep sedation and spontaneous ventilation [8–10]. Nowadays, multiple bronchoscopic interventions are developed to remove airway obstructions. Therefore, general anesthesia with HFJV and special monitoring are necessary for these procedures.

The incidence of hypercapnia in our study was 93.2%, in which 14.6% of patients had severe hypercapnia. This finding was 26.4%–53.4% higher than other reports [10,11]. High complexity of multiple bronchoscopic interventions, poor patient general conditions, and severe bronchial stenosis can contribute to high incidences of hypercapnia. Lobectomy, dynamic airway collapse, lateral ventilation, working instruments, bleeding, and local spraying anesthetics can lead to hypoventilation and hypercapnia.

The incidence of hypoxemia in our study was 2.2%, which is lower than the 3.7%–23.2% reported by other research [10,11]. Our lower findings can be accounted for by our use of FiO_2_ 100% compared with the lower concentrations of FiO_2_ used in other studies. Some reports recommend an inhaled FiO_2_ level below 40% to reduce the risk of combustion during the laser coagulation procedure [12,13]. For our patients, bronchoscope was intentionally kept away from APC during laser therapy. Furthermore, jet ventilation was delivered through the lateral port, and the oxygen concentration below the stenosis trachea was significantly lower than FiO_2_ [14]. Through these methods, the fire risk of the high concentration of FiO_2_ was effectively prevented.

Our study found that severe acute hypercapnia was associated with increased postoperative cardiac failure risks and serum glucose levels, as well as lengthy extubation and longer operation room stays. Violent variations in PaCO_2_ significantly affect sympathetic nerve and systemic hemodynamics [15]. Hypercapnia can suppress myocardial contractility by causing progressive intracellular acidosis [16,17], which can be counter balanced by the effects of CO_2_ on the central and autonomic systems [16]. The high incidence of postoperative cardiac failure (4.3%, 14/323) in our study can be attributable to a combination of hypercapnia and generally poor patient conditions.

Our study revealed that mild to moderate hypercapnia (PaCO_2_:61–100 mmHg) do not delay recovery, which is consistent with other studies [18–20]. Hypercapnia can lead to a shorter recovery period from an operation using inhaled anesthetics, possibly by enhancing respiratory drive and promoting inhaled anesthetic removal from the lungs [18,20]. Furthermore, a recent study demonstrated the neuroprotective effects of mild to moderate hypercapnia (PaCO_2_:61–100 mmHg) in rat models with transient cerebral ischemia reperfusion injury. However, severe hypercapnia (PaCO_2_: >100 mmHg) may cause brain injury by aggravating brain edema [5]. Fortunately, no neurologic deficits and cerebral hemorrhages occurred in our patients. Basing on the above evidence, we believe that acute and transient CO_2_ retention, lower than 100mmHg, are unlikely to cause any serious consequences in patients receiving bronchoscopic interventions under general anesthesia when arterial oxygen desaturation is avoided. Further animal and clinical studies are necessary to elucidate the diverse effects of hypercapnia on myocardium acidosis and on neuron protection during general anesthesia.

This study presents several limitations. First, this is a retrospective chart review study, and the group assignment was based on BGA level without randomization. The BGA sampling was not controlled, and the group assignment might be biased. Second, the carbon dioxide levels were obtained from ABG analysis and not from trans-cutaneous monitoring system or end-tidal PCO_2_ measurements. Blood samples with the highest carbon dioxide values were analyzed to evaluate the effects of hypercapnia on surgical outcomes. Third, though more than 300 patients were included in our study, only 43 surgery related complications occurred and were included in multiple regression analysis. Future studies with larger sample size are needed to further enhance our conclusions. Fourth, our conclusion was drawn from patients with bronchoscopic interventions under general anesthesia. Therefore, this conclusion should not be extrapolated to other patients who receive cardiovascular, neurologic, or abdominal surgeries.

In conclusion, our study demonstrates that acute moderate CO_2_ retention may not associated with serious consequences among patients receiving bronchoscopic interventions under general anesthesia.
